# Management of Large Non-Pedunculated Polyps of the Colon: Practice-Oriented Answers to Clinical Questions

**DOI:** 10.3390/jcm15030929

**Published:** 2026-01-23

**Authors:** Cecilia Capelli, Alberto Gattuso, Luigi Tuccillo, Marco Di Marco, Leonardo Frazzoni

**Affiliations:** 1 Department of Medical and Surgical Sciences, University of Bologna, 40138 Bologna, Italy; cecilia.capelli3@studio.unibo.it (C.C.); alberto.gattuso@studio.unibo.it (A.G.); luigi.tuccillo@studio.unibo.it (L.T.); 2 Gastroenterology and Digestive Endoscopy Unit, Rimini Hospital, AUSL Romagna, 47923 Rimini, Italy; marco.dimarco@auslromagna.it

**Keywords:** large non-pedunculated colorectal polyps, endoscopic mucosal resection, endoscopic submucosal dissection, colorectal neoplasms, submucosal invasion, laterally spreading tumor, optical diagnosis, colonoscopy

## Abstract

**Background/Objectives**: Large (≥20 mm) non-pedunculated colorectal polyps (LNPCPs) are challenging lesions with a variable, yet non-negligible risk of advanced neoplasia. While correct management is therefore mandatory, a discrepancy often persists between guideline recommendations and daily endoscopic practice. To bridge this gap, we performed a comprehensive and structured review of the available evidence, aiming to synthesize the current knowledge and provide practice-oriented guidance for the optimal management of LNPCPs throughout the diagnostic–therapeutic pathway. **Methods**: A comprehensive literature review was independently performed. We systematically searched PubMed and Google Scholar up to December 2025. After the literature review, we identified the most clinically relevant and controversial aspects in the endoscopic management of LNPCPs. These key areas were then translated into focused, practice-oriented clinical questions. **Results**: We formulated 14 practice-oriented questions addressing the key steps of endoscopic management of LNPCPs. These questions cover the entire diagnostic–therapeutic pathway, including lesion detection, morphological characterization, optical diagnosis and risk stratification for submucosal invasion, selection of the optimal resection technique, and post-resection surveillance strategies. For each question, the current evidence was synthesized to provide concise, clinically applicable answers aimed at supporting real-world endoscopic decision-making. **Conclusions**: The endoscopic management of LNPCPs requires a structured and evidence-based approach that integrates accurate assessment, appropriate technique selection, and tailored post-resection surveillance. By framing current evidence into focused, practice-oriented questions, this review aims to bridge the gap between guideline recommendations and real-world endoscopic practice. The proposed framework may support endoscopists in daily clinical decision-making, promoting the appropriate use of advanced endoscopic techniques and ultimately improving patient outcomes.

## 1. Introduction

Large non-pedunculated colorectal polyps (LNPCPs), defined as sessile or flat lesions ≥ 20 mm in size, represent a significant challenge in modern endoscopic practice. The prevalence of LNPCPs varies according to the clinical setting, differing markedly between primary screening colonoscopy and diagnostic follow-up after a positive stool test. While LNPCPs constitute a minority of findings in average-risk populations (accounting for approximately 1–2% of all polyps), they impose a disproportionate clinical burden. Notably, in screening programs based on the fecal immunochemical test, the detection rate of these lesions rises significantly, approaching 1 in every 24–25 colonoscopies [1,2]. Consequently, LNPCPs are not merely incidental findings but distinct clinical entities that often require management by expert endoscopists.

Accurate recognition, morphological characterization, and appropriate risk stratification are pivotal, as LNPCPs harbor a substantial risk of advanced neoplasia, with the prevalence of covert submucosal invasive cancer being estimated at approximately 8–10% in large multicenter cohorts [3]. Although international guidelines offer structured recommendations for detection, optical diagnosis, and therapeutic strategy, real-world management is frequently compromised by variability in assessment, operator-dependent decision-making, and the inconsistent adoption of validated classification systems. To address these gaps, we conducted a comprehensive review of the current evidence, aiming to synthesize available knowledge and provide practice-oriented guidance for the optimal management of LNPCPs throughout the diagnostic and therapeutic pathway.

## 2. Materials and Methods

We performed a narrative review based on a comprehensive literature search, independently conducted by three authors (C.C., A.G., L.T.). We systematically searched PubMed and Google Scholar up to December 2025 to identify studies evaluating various aspects of LNPCPs, including detection, characterization, predictors of submucosal invasive cancer, technical difficulty of resection, outcomes of endoscopic mucosal resection (EMR) and endoscopic submucosal dissection (ESD), and post-resection surveillance.

The PubMed search was performed using a combination of free-text keywords and Medical Subject Headings (MeSH): (“Colonic Polyps” [MeSH] OR “Colorectal Neoplasms” [MeSH] OR “large non-pedunculated colorectal polyp” OR “LNPCP” OR “laterally spreading tumor” OR “LST” OR “large sessile polyp” OR “flat colorectal lesion”) AND (“Endoscopic Mucosal Resection” [MeSH] OR “Endoscopic Submucosal Dissection” [MeSH] OR “endoscopic resection” OR “EMR” OR “ESD”) AND (“classification” OR “risk stratification” OR “optical diagnosis” OR “submucosal invasion”).

The Google Scholar search was intentionally broader to maximize sensitivity. The terms “large non-pedunculated colorectal polyp”, “laterally spreading tumor”, “endoscopic mucosal resection”, “endoscopic submucosal dissection”, “LST-G/N-G”, “morphological classification”, “submucosal invasive cancer”, and “optical diagnosis” were used in multiple combinations. Given the platform’s non-curated indexing, the first several hundred titles were screened manually to identify relevant studies.

Inclusion criteria comprised systematic reviews and meta-analyses, clinical studies, and large case series on patients with LNPCPs; only full-text articles were considered.

Exclusion criteria included case reports or small case series (<5 patients), narrative reviews without original data, editorials, letters, and studies involving non-human models.

**Figure 1:**
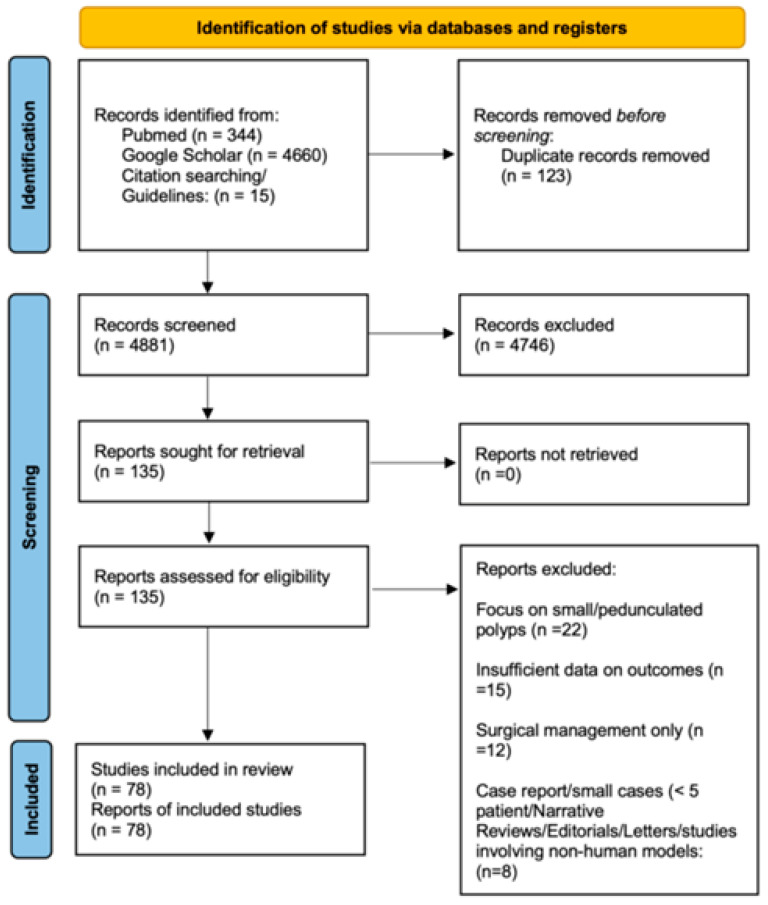
Flowchart of study selection.

No language or publication date restrictions were applied. Additionally, the reference lists of included studies were manually screened for further eligible articles. The detailed study selection process is illustrated in the flowchart in Figure 1.

Following the literature review, we identified the most clinically relevant and controversial aspects in the management of LNPCPs. These key areas were translated into focused, practice-oriented clinical questions reflecting real-world decision-making. Each question was addressed by critically appraising the existing evidence and integrating it with current guideline recommendations and expert interpretation, with the aim of providing pragmatic and applicable answers for daily clinical practice.

## 3. Results

We formulated 14 practice-oriented questions addressing the key steps of endoscopic management of LNPCPs. These questions span the entire diagnostic–therapeutic pathway, including lesion detection and morphological characterization, optical diagnosis and risk stratification for submucosal invasion, selection of the most appropriate resection technique, and post-resection surveillance strategies. For each question, available evidence was synthesized to provide concise, clinically applicable answers, designed to support real-world endoscopic decision-making. Questions and answers are summarized in Table 1.

### 3.1. How Should a Large Non-Pedunculated Colorectal Polyp Be Correctly Detected?

Accurate recognition and characterization of LNPCPs constitute the cornerstone of appropriate therapeutic decision-making. As the diagnostic accuracy is contingent upon mucosal visibility, detection depends on a high-quality endoscopic examination; indeed, even the most skilled endoscopists cannot assess lesions that they cannot clearly visualize. Adherence to quality standards defined by the European Society of Gastrointestinal Endoscopy (ESGE) is therefore mandatory [4,5]. Accurate inspection requires a cecal intubation rate > 90%, an adequate withdrawal time (mean > 6 min), and meticulous cleansing to ensure adequate bowel preparation. Cecal intubation is a prerequisite for complete visualization of the colorectum and must be confirmed with photo or video documentation.

Furthermore, clear cecal image documentation is associated with a higher polyp detection rate [6]. Colonoscope withdrawal time serves as a proxy for the time dedicated to mucosal inspection. A mean withdrawal time exceeding 6 min is associated with a significantly higher adenoma detection rate (ADR). Large-scale analyses have confirmed this positive correlation, demonstrating a 3.6 absolute increase in ADR for every minute increase in withdrawal time [7]. As emphasized by ESGE guidelines, the quality of bowel preparation is intrinsically linked to these performance measures, specifically ADR and cecal intubation rates [8]. Consequently, in cases of inadequate bowel preparation, the procedure must be rescheduled to ensure a high-quality examination rather than proceeding with a suboptimal inspection [9].

To further optimize diagnostic yield, particularly for subtle right-sided lesions, endoscopists should employ dynamic maneuvers such as cecal retroflexion or a “second-look” examination, which effectively mitigates the risk of missing lesions located behind haustral folds [10,11]. Similarly, the adoption of water exchange or water immersion techniques has demonstrated superiority over standard gas insufflation in detecting serrated and flat lesions, primarily by enhancing mucosal cleanliness and minimizing colonic spasm [12]. Finally, the integration of artificial intelligence (AI) represents a rapidly evolving frontier in quality assurance; computer-aided detection (CADe) systems serve as an automated “second observer,” reducing perceptual errors and standardizing the recognition of LNPCPs [13].

### 3.2. What Are the Common Mistakes in the Initial Assessment of Large Non-Pedunculated Colorectal Polyps?

Despite the widespread adoption of advanced endoscopic imaging, standardized classification systems, and international guidelines, diagnostic errors in lesion assessment persist in daily practice. The initial assessment is based on the accurate evaluation of size and morphology, as these factors dictate communication of findings, the stratification of covert submucosal cancer risk, and the selection of appropriate resection techniques [14,15]. Precise size estimation of LNPCPs is critical for optimal clinical decision-making; however, measurement inaccuracy remains a pervasive and often underestimated issue. A recent large prospective cohort study demonstrated that unassisted endoscopic size estimation is accurate (within a 5 mm margin) in only 53.4% of cases. This discrepancy is frequently driven by “terminal digit bias,” a phenomenon where endoscopists subconsciously round estimates to the nearest preferred digit—most commonly 0 (65.2%) or 5 (30.0%) [16]. Since the depth of submucosal invasion (SMI) increases with lesion size, underestimation can lead to the selection of inappropriate therapeutic techniques [17]. To mitigate these errors, endoscopists must employ objective reference standards during the assessment. While tools like biopsy forceps or polypectomy snares allow for calibration in smaller lesions, their utility is limited for lesions > 20 mm. Therefore, the most promising approach for large lesions relies on advanced digital systems. In particular, the virtual scale endoscope (VSE) and AI-based platforms offer real-time in vivo measurements, overcoming issues of perspective and distortion [18].

Morphological characterization, a crucial predictor of neoplastic behavior, is standardized according to the Paris Classification. This system categorizes lesions by their elevation or depression relative to the mucosal plane—such as sessile (0-Is), slightly elevated (0-IIa), flat (0-IIb), or slightly depressed (0-IIc)—providing initial insight into the risk of colorectal cancer (CRC) [19]. Despite its pivotal role, interobserver agreement regarding the Paris classification remains suboptimal, irrespective of the endoscopist’s training level, sex, or specialty, as shown in a recent prospective video-based study by R. Djinbachian et al. [15]. Discrepancies often arise from the difficulty in distinguishing between specific subtypes (e.g., 0-IIa vs. 0-IIb). To address this, a simplified system categorizing lesions as either polypoid (pedunculated/sessile) or non-polypoid (flat/depressed/slightly elevated) has been proposed, significantly increasing global diagnostic accuracy to 91.6% [20].

### 3.3. When Should Deep Submucosal Invasion Be Suspected in a Large Non-Pedunculated Colorectal Polyp?

Deep SMI, defined in the colorectum as invasion extending ≥1000 µm into the submucosal layer, is the critical determinant of therapeutic strategy. It differentiates lesions that are amenable to curative endoscopic resection from those requiring surgery due to the high risk of lymph node metastasis [21]. Morphologically, surface depression is the strongest independent predictive factor for SMI. A recent decision-tree analysis has shown that the prevalence of deep SMI in depressed LNPCPs was approximately 62%, compared to significantly lower rates in flat or nodular lesions without depression [22]. Based on their morphology, LNPCPs are stratified into granular (LST-G) and non-granular (LST-NG) subtypes. The LST-NG morphology is associated with a heightened risk of SMI, which may exceed 30–40% in pseudo-depressed lesions (LST-NG-PD) ≥ 20 mm; conversely granular types (LST-G) typically harbor invasion only within large dominant nodules (LST—granular mixed type, LST-GM). Notably, a seminal study by Burgess et al. established these risk profiles, clearly delineating the risk of SMI according to LST classification [3,23]. Beyond classification subtypes, specific overt features that are visible under white light are highly specific for deep SMI. The presence of ulceration raises the probability of deep invasion to approximately 75%, implying that the tumor has disrupted mucosal integrity or outgrown its blood supply [24]. Fold convergence, the “puckering” of colonic folds toward the lesion, indicates a desmoplastic reaction in the submucosa, caused by the tumor infiltrating deeper layers and contracting the surrounding tissue [25]. Similarly, spontaneous bleeding, or friability that leads to bleeding without significant trauma, suggests neovascularization and tissue fragility associated with malignancy.

The “non-lifting sign” (NLS)—defined as the failure of a lesion to elevate following submucosal fluid injection—demonstrates high specificity (>95%) but low sensitivity (<70%) for deep SMI in treatment-naïve lesions. While its presence is strongly predictive of invasive carcinoma, its absence does not reliably exclude malignancy, particularly in invasive cancers associated with minimal desmoplastic reaction [26]. Furthermore, in LST-NG lesions, a positive NLS requires cautious interpretation; these lesions may exhibit poor lifting due to intrinsic fibrosis rather than malignant infiltration, potentially mimicking invasive behavior [27].

Virtual chromoendoscopy (VCE) technologies enhance the contrast of mucosal surface patterns and vasculature, allowing for a detailed “optical biopsy” that correlates strongly with histology. Under VCE (NBI/BLI), NICE Type 3 and JNET Type 3 patterns—characterized by an amorphous surface pattern and interrupted, thick vessels—are highly specific indicators of deep SMI that generally necessitate surgical referral. The equivocal JNET Type 2B pattern represents a diagnostic “gray zone” requiring adjudication via dye-based magnifying chromoendoscopy. The Kudo type Vn (non-structural) pit pattern serves as the definitive contraindication to endoscopic resection, confirming deep invasion with high specificity [28].

### 3.4. Should Large Non-Pedunculated Colorectal Polyps Be Biopsied or Is It Better to Avoid Sampling?

Current international guidelines strongly advise against the routine biopsy of LNPCPs prior to referral for resection. The traditional practice of preliminary tissue sampling is now considered counterproductive due to two primary reasons: diagnostic inaccuracy and technical hindrance [24].

Forceps biopsies are limited by significant sampling errors due to the histological heterogeneity of large lesions. This limitation was highlighted by a recent retrospective analysis involving 586 LNPCPs: comparison with final EMR specimens revealed significant histological discordance, demonstrating that 26.1% of lesions were up-staged and 13.8% down-staged following complete resection [29]. Consequently, a “benign” biopsy result may provide false reassurance and should not influence the therapeutic strategy. Secondly, and perhaps most importantly, biopsy induces mechanical injury leading to submucosal fibrosis, often resulting in a positive NLS. In this context, the NLS reflects iatrogenic scarring rather than malignant infiltration, thereby increasing the technical difficulty, the likelihood of incomplete resection or recurrence, and the risk of perforation [30]. Therefore, biopsy should be reserved exclusively for lesions where optical features strongly suggest deep SMI (e.g., JNET Type 3, Kudo V), where histological confirmation is required prior to surgical referral. For all other lesions, the polyp should remain treatment-naïve to maximize the probability of a curative endoscopic outcome [31].

### 3.5. When Should a Large Non-Pedunculated Colorectal Polyp Be Scheduled in a Dedicated Therapeutic Session Instead of a Standard List?

The increasing detection of LNPCPs challenges the traditional “see-and-treat” model within standard 45 min diagnostic slots. In this constrained environment, endoscopists face a critical dilemma: attempting a rushed, unplanned resection risks incomplete excision and adverse events, while deferring with biopsy typically induces fibrosis that compromises future therapy. This paradigm is addressed by major international frameworks, including the ESGE and BSG guidelines, which strongly recommend that complex lesions be managed exclusively in settings that guarantee the requisite expertise and resource allocation [4,24,32].

A prevalent error in daily clinical practice is the attempt to resect lesions that are larger than 20 mm during standard diagnostic slots. The current ESGE guidelines mandate high-quality standards that are inherently time-consuming, such as dynamic submucosal injection, controlled piecemeal resection, thermal ablation of post-EMR margins, and prophylactic clipping in the right colon. Attempting these maneuvers under time pressure is an independent risk factor for the incomplete resection rate (IRR), which can exceed 15–20% in suboptimal settings. The duration of the procedure is directly correlated with the presence of residual neoplasia at follow-up; therefore, if the estimated resection time—including the management of potential intraprocedural adverse events—exceeds the allocated slot, rescheduling to a dedicated session is mandatory to ensure a meticulous and curative procedure [24,33].

Nevertheless, the decision to reschedule an LNPCP is often met with resistance due to perceived inefficiency. However, current analyses demonstrate that the “see-and-treat” approach for complex lesions generates a substantial hidden “cost of failure.” Economic modeling has established that endoscopic resection maintains its cost-effectiveness advantage over surgery only when adverse event rates remain below 12% and technical success exceeds 75%. Attempting resection under time pressure significantly risks breaching these thresholds, leading to expensive surgical salvage or the management of recurrences. Furthermore, a complication in a standard list triggers a “domino effect” of delays and cancelations, generating indirect costs that far exceed the expense of a planned second procedure where prophylactic measures (e.g., clipping) and proper resection techniques can be applied cost-effectively [34,35].

### 3.6. How to Predict the Technical Difficulty of Large Non-Pedunculated Colorectal Polyp Resection?

Reliance on subjective assessment to predict the technical difficulty of LNPCPs is notoriously unreliable, particularly given the pervasive tendency to misestimate the actual size of the lesions. Accurate size measurement is paramount, as it constitutes the most heavily weighted variable within the validated SMSA (Size, Morphology, Site, Access) scoring system [36]; indeed, increasing size correlates linearly with both technical complexity and the risk of covert submucosal invasion. To mitigate this uncertainty, endoscopists should utilize the SMSA score to objectively guide procedural planning. While smaller lesions yielding scores < 10 (Level 1–2) are typically manageable in standard diagnostic slots, an SMSA score ≥ 10 represents a critical management threshold. Large lesions resulting in Level 3 scores (10–12) are associated with unpredictable procedural durations; scheduling them in standard lists risks compromising the resection quality (e.g., inadequate margin ablation) due to time constraints, thus warranting a dedicated therapeutic session. Lesions with scores > 12 (Level 4) constitute an “extreme complexity” subgroup, associated with incomplete resection rates exceeding 40%. These cases frequently necessitate advanced techniques (e.g., ESD, hybrid resection) or specific adjuncts that are unavailable in standard settings, rendering a “see-and-treat” approach contraindicated and referral to an expert center mandatory [36].

Finally, decision-making for Level 4 lesions or suspected deep SMI should not be solitary. A brief discussion in a multidisciplinary team (MDT) is recommended, as it has been shown to alter the management plan in up to 20% of cases, preventing futile attempts or unnecessary surgery [37].

Technical complexity is frequently exacerbated by anatomical challenges compromising stability and access. Lesions involving the appendiceal orifice or ileocecal valve are particularly hazardous due to the thinness of the cecal wall and limited maneuverability, often requiring expert management or advanced techniques like endoscopic full-thickness resection (EFTR). Similarly, lesions that are concealed behind haustral folds or at flexures present significant access difficulties, often necessitating retroflexion or cap-assisted stabilization. Special caution is required for lesions extending into a diverticulum, where the absence of muscularis propria drastically increases the perforation risk, often contraindicating standard EMR. Finally, involvement of the dentate line presents unique challenges including pain, increased bleeding risk due to dual systemic venous drainage, and scope instability. Maintaining a stable scope position remains a prerequisite for safety, as instability independently increases the risk of adverse events and incomplete resection [24,25].

Table 2 summarizes the recommended management of LNPCPs according to predicted complexity of endoscopic resection.

### 3.7. What Level of Expertise Is Required to Safely Manage Large Non-Pedunculated Colorectal Polyps?

Safe management of LNPCPs requires specific cognitive and technical competencies that extend significantly beyond standard diagnostic colonoscopy. While initial competence is generally established after a minimum of 30 independent EMR procedures performed under supervision, this threshold represents only the entry point for independent practice rather than mastery. To maintain expert status and ensure quality assurance, endoscopists must sustain a high annual case volume; European guidelines recommend a minimum of 70 independent resections of lesions ≥ 20 mm per year. Expertise is further characterized by the achievement of specific key performance indicators (KPIs); a proficient endoscopist should achieve technical success—defined as complete resection in a single session—in at least 91% of cases [17].

Long-term efficacy is benchmarked by an adenoma recurrence rate at first surveillance of less than 16%. Notably, with the routine application of margin thermal ablation (MTA), this benchmark tightens significantly to <2.6%. Regarding safety, accepted minimum standards require maintaining intraprocedural bleeding rates below 19% and perforation rates below 5%, with desirable expert targets of ≤14% and ≤3.5%, respectively [17].

Beyond quantitative metrics, the expert profile requires mastery of cognitive skills for accurate optical diagnosis using validated classifications (e.g., JNET, NICE). This is essential to stratify the risk of submucosal invasion and appropriately select between *en bloc* resection, piecemeal EMR, or surgical referral. Furthermore, expertise encompasses the autonomous ability to manage intraprocedural adverse events and interpret histopathological findings to guide surveillance [17,38]. Moreover, while standardized compact training (e.g., e-learning combined with hands-on workshops) has been shown to reduce recurrence rates by up to 50% for lesions sized 20–39 mm, this protective effect is attenuated for lesions ≥ 40 mm. This suggests that for very large and complex lesions, short-term training is insufficient in the absence of high annual case volumes [39].

### 3.8. When Is EMR the Preferred Approach for Large Non-Pedunculated Colorectal Polyps?

In the management of LNPCPs, EMR is the preferred therapeutic approach for the vast majority of lesions in Western practice. It is efficient, widely available, and possesses a robust safety profile [40]. The clinical decision to prioritize EMR is predicated on the assessment of malignancy risk. The primary limitation of EMR, particularly for LNPCPs, is the frequent necessity for piecemeal resection (p-EMR). This approach complicates the histological evaluation of the deep and lateral margins of the specimen as a single unit, making it challenging—and sometimes impossible—to accurately stage the depth of submucosal invasion (T1b vs. T1a) or to confirm curative (R0) resection in the context of malignancy [41]. Consequently, EMR is only the preferred approach when the risk of SMI is negligible, and when the lesion is predicted to be benign or to harbor only high-grade dysplasia [24].

LST-G homogeneous (LST-GH) lesions carry a submucosal invasion risk of <1% [3]. In these cases, *en bloc* resection for oncological staging is considered unnecessary, and p-EMR is the technique of choice given the negligible risk of transecting an invasive focus during the fragmentation of the lesion [24]. LST-NG flat elevated (Paris 0-IIa) lesions have an intermediate invasion risk (~6%) and are also suitable for EMR, particularly for lesions < 20–30 mm, where *en bloc* snare resection is feasible [42]. For larger lesions, p-EMR is acceptable, provided that high-definition optical diagnosis confirms the absence of depression or amorphous patterns [42].

Conversely, LST-GM nodular mixed-type lesions comprising a mixture of granules and larger, dominant nodules (often > 10 mm) exhibit a higher risk of SMI, estimated at approximately 12% [42]. Crucially, as invasion typically occurs beneath the largest dominant nodule, and EMR remains a preferred approach only if the dominant nodule can be resected *en bloc* or if it displays benign optical features (e.g., JNET 2A, Kudo IV). In this scenario, a targeted EMR strategy, wherein the large nodule is removed first in one piece followed by piecemeal resection of the granular component, is a validated approach [24].

### 3.9. In Which Situations Is ESD the Most Appropriate Technique for Large Non-Pedunculated Colorectal Polyps?

The current ESGE guidelines advocate for “selective ESD”, a strategy aiming to balance oncological safety with cost-effectiveness, by reserving ESD for lesions where EMR is deemed technically unfeasible or associated with inferior oncological outcomes [24,43]. For LNPCPs, the primary oncological indication for ESD is the pre-procedural suspicion of superficial submucosal invasive carcinoma (i.e., T1a). In this clinical setting, ESD is the mandatory therapeutic strategy to achieve an *en bloc* R0 resection, allowing for precise pathological evaluation by accurately measuring the depth of SMI and assessing lymphovascular involvement. These two parameters are the critical determinants in establishing whether the endoscopic procedure is curative or if surgical rescue is required [41].

Consequently, the identification of specific optical and morphological features constituting absolute indications for ESD is paramount. Under VCE, a JNET Type 2B classification serves as a validated predictor of high-grade dysplasia or superficial SMI, necessitating the precise dissection plane that ESD provides [28]. Similarly, the identification of a Kudo pit pattern Vi correlates strongly with early invasive neoplasia, reinforcing the requirement for the controlled, single-piece resection provided by ESD [44].

From a morphological perspective, specific subtypes of LSTs represent independent indications for ESD due to their aggressive biological behavior. Notably, LST-NG-PDs larger than 20 mm are associated with a high risk of SMI [45]. Furthermore, lesions presenting with a Paris 0-IIc component are characterized by rapid vertical growth and multifocal invasion [2]. For these high-risk morphologies, ESD is the standard of care to ensure the complete removal of the invasive front and achieve oncological radicality. In the context of the LST-GM nodular mixed type, if the dominant nodule is excessively large or exhibits signs of invasion (i.e., JNET 2B), ESD is mandatory [24].

### 3.10. Is There a Role for Hybrid ESD or Alternative Techniques for Large Non-Pedunculated Colorectal Polyps?

The therapeutic landscape for LNPCPs has expanded beyond the traditional binary choice between EMR and ESD. Hybrid and simplified techniques have been developed to bridge the gap between the efficiency of EMR and the oncologic precision of ESD. These strategies aim to mitigate the technical hurdles hindering widespread ESD adoption in the West, specifically the steep learning curve, prolonged procedure times, elevated costs, and incidence of severe adverse events [46]. Hybrid ESD (H-ESD) combines an initial circumferential incision and partial submucosal dissection with a final snare resection. Although H-ESD significantly reduces procedure duration and perforation risk compared to conventional ESD (C-ESD), comprehensive meta-analyses indicate inferior oncological outcomes for colorectal lesions, characterized by lower *en bloc* and R0 resection rates and increased local recurrence risks [46]. Furthermore, a distinction must be made between intentional H-ESD and rescue H-ESD, wherein the procedure is initiated as a conventional ESD but converted intra-operatively to a hybrid snare resection due to technical difficulties. In contrast to intentional H-ESD, rescue H-ESD is a salvage maneuver and typically performed under suboptimal conditions, naturally carrying lower success rates [47]. In this context, Yamaguchi et al. investigated the comparative efficacy of these techniques across 364 colorectal ESD procedures, demonstrating that the *en bloc* resection rate increased significantly in the order of rescue H-ESD (48.6%), intentional H-ESD (78.3%), and C-ESD (97.7%) (*p* < 0.001) [48]. Consequently, H-ESD should be reserved for trainees during their learning curve or as a rescue technique for difficult cases rather than as a primary strategy for lesions with suspected superficial submucosal invasion [49].

While H-ESD represents a simplification of ESD, underwater EMR (U-EMR) offers a distinct paradigm by fundamentally altering the resection environment. By replacing luminal gas with water, U-EMR obviates the need for submucosal injection, leveraging fluid mechanics to facilitate resection [50]. Current ESGE guidelines recommend U-EMR as a valid alternative to conventional EMR for LNPCPs [24]. The benefit is most pronounced for “medium–large” lesions (20–30 mm), where U-EMR achieves significantly higher *en bloc* rates and lower recurrence rates (3.4% for U-EMR vs. 13.1% for C-EMR). This suggests that for this specific size cohort, U-EMR effectively bridges the gap between EMR and ESD, allowing for *en bloc* removal without the complexity of dissection.

Conversely, mechanical modifications have been developed to streamline C-ESD itself. Traction-assisted ESD (T-ESD) involves applying devices (i.e., clips with rubber bands, silk threads, etc.) to the lesion’s flap to exert tension, thereby creating traction or counter-traction [51]. This facilitates improved visualization of the submucosal space and accelerates resection [52]. Evidence suggests that T-ESD not only reduces procedure time and perforation risk but also substantially flattens the learning curve, enabling less experienced endoscopists to achieve expert-level efficiency and R0 resection rates [52].

Similarly, the pocket-creation method (PC-ESD) is another simplified strategy that does not require external devices. It involves creating a submucosal tunnel (pocket) beneath the lesion before completing the circumferential incision. The overlying mucosa acts as a “roof,” stabilizing the endoscope and providing natural traction. PC-ESD has been shown to improve *en bloc* resection rates and reduce procedure time, functioning effectively as a “built-in” traction method [53]. Finally, these techniques may be combined to perform pocket-creation with traction ESD (PT-ESD), where clips may be used to apply traction to the tissue, facilitating the opening of the mucosal pocket [54].

### 3.11. How Much Does Lesion Location Influence the Choice of Resection Strategy?

Lesion location is a critical determinant in the therapeutic algorithm for LNPCPs. The choice of the most appropriate resection technique is heavily influenced by the anatomical segment [24]. In the rectum, the potential morbidity of surgical interventions—specifically the risk of a permanent stoma—strongly favors ESD. The extraperitoneal anatomical setting mitigates the clinical consequences of perforation, while luminal stability facilitates *en bloc* resection, making ESD the preferred strategy to maximize organ preservation. Conversely, in the proximal colon, the strategy is constrained by the significantly thinner muscularis propria, which lowers the threshold for deep mural injury. In this segment, ESD is technically demanding and should be strictly reserved for non-granular LSTs or lesions with clear suspicion of carcinoma, where *en bloc* resection is mandatory [55,56].

Luminal gravity and vascular microanatomy play a pivotal role in selecting the resection technique. U-EMR is most effective in “gravity-dependent” positions, where water naturally pools, magnifying the view and stabilizing the colon wall. In “non-dependent” positions, maintaining the water interface can be challenging, often necessitating dynamic patient repositioning or conversion to standard gas-insufflated EMR [57]. Lesions located on the mesenteric side are associated with larger perforating vessels. Resection in this area, particularly with ESD, carries a higher risk of immediate and delayed bleeding. Prophylactic coagulation of visible vessels (coagulation grasping) is mandatory in these locations. Conversely, the antimesenteric wall is often thinner, requiring extreme caution during submucosal injection and snaring to avoid full-thickness injury [58].

Finally, specific anatomical landmarks—namely the appendiceal orifice (AO), ileocecal valve (IV), colonic flexures, and the dentate line—represent “difficult locations”, where the resection strategy is dictated by technical feasibility rather than histology alone. In these areas, maneuverability is frequently compromised by luminal instability and unfavorable access angles. Polyps involving the AO are technically challenging due to the lack of submucosa and the risk of “tunneling” into the appendix. Standard injection can paradoxically close the lumen, rendering access impossible. U-EMR is increasingly preferred as the first-line technique; the water keeps the lumen open and floats the polyp tissue out of the orifice [59]. The IV is characterized by fatty infiltration (lipomatosis), which prevents effective lifting, and poor access; therefore, cap-assisted EMR or U-EMR are recommended. The cap allows the endoscopist to depress the valve folds and expose the ileal aspect of the lesion, while U-EMR is particularly useful, as it does not require lifting, thus bypassing the issue of lipomatosis [60]. Polyps extending into a diverticulum lack a muscular layer at the base (only mucosa and serosa), creating an extreme perforation risk. Injection can invert the diverticulum, obscuring the polyp. In this context, U-EMR is the technique of choice. Water immersion creates a pressure gradient that can evert the diverticulum, exposing the polyp for safe snare capture without needle injection [61]. The dentate line presents challenges related to somatic innervation and hemorrhoidal drainage. Resection here requires local anesthetic injection (e.g., lidocaine/epinephrine) to prevent severe pain and facilitate hemostasis. Endoscopists must also be prepared to manage brisk arterial bleeding from hemorrhoidal vessels using coagraspers or clips [62].

### 3.12. What Are the Most Frequent Technical Errors During Endoscopic Resection of Large Non-Pedunculated Colorectal Polyps?

A fundamental initial error lies in the improper selection of the endoscopic resection technique. The indiscriminate application of piecemeal EMR (p-EMR) to lesions with suspected SMI compromises accurate staging and curative potential. Conversely, unwarranted use of ESD for benign lesions increases perforation risks and prolongs procedural time without yielding distinct clinical benefit [41,42]. During C-EMR, static injection often results in a steep, dome-like configuration; this geometry causes the snare to slip off the polyp, leading to superficial and inefficient resections [63]. This technical pitfall increases procedure time, fragmentation, and the risk of leaving residual tissue islands—factors that are directly correlated with higher recurrence rates [64]. To mitigate this, dynamic injection is recommended to generate a flat plateau that facilitates stable snare seating [65]. Furthermore, relying exclusively on normal saline for large lesions is suboptimal due to its rapid dissipation. Attempting resection in a diminished submucosal cushion significantly heightens the risk of muscle entrapment and perforation [65]. Current guidelines and recent studies advocate for the use of viscous injection solutions (e.g., succinylated gelatin, hydroxyethyl starch, or glycerol) for large lesions. These agents maintain the submucosal cushion for a significantly prolonged time, enhancing resection efficiency and reducing technical complexity [38].

During the resection phase, technical failure often stems from a mismatch between snare properties (e.g., monofilament vs. multifilament; symmetrical vs. asymmetrical) and polyp morphology. A snare lacking sufficient radial force may fail to open flat against a rigid LNPCP, leading to transection through the mucosal layer rather than the submucosal plane and leaving residual adenoma at the base [66]. Conversely, employing an excessively large snare for piecemeal resection within a narrow lumen (e.g., the sigmoid colon) increases the risk of contralateral wall contact or inadvertent capture of haustral folds, potentially causing thermal injury or full-thickness resection [66]. Furthermore, the omission of MTA is a major driver of recurrence. Evidence strongly supports the application of snare tip soft coagulation (STSC) on the resection margin during p-EMR, which significantly reduces adenoma recurrence by eradicating microscopic residual tissue [24].

Regarding ESD, blind coagulation—the activation of thermal energy within pooled blood without vessel visualization—remains a leading cause of delayed perforation, secondary to transmural thermal injury [67]. Additionally, technical errors frequently stem from incorrect electrosurgical settings. A common pitfall is the use of EndoCut Q (optimized for snare resection) instead of EndoCut I (optimized for needle knives) during the incision phase, which may lead to excessively aggressive cutting [68]. In both EMR and ESD, failure to employ dynamic patient positioning represents a preventable error. Leaving a lesion in a gravity-dependent position causes fluid pooling and poor visualization. Rotating the patient to leverage gravity provides natural traction and maintains a clear endoscopic field [21]. Finally, errors in defect closure often result from inadequate bleeding risk stratification. Current guidelines support complete prophylactic clipping for right-sided lesions to prevent delayed bleeding, whereas routine clipping in the left colon is generally unnecessary and not cost-effective [24].

### 3.13. How Should Non-Lifting Large Non-Pedunculated Colorectal Polyps Be Managed?

Historically, the NLS was considered a pathognomonic indicator of deep SMI and a contraindication to endoscopic resection. However, contemporary evidence clarifies that the NLS is not specific to malignancy and frequently arises from benign iatrogenic fibrosis [69]. In the context of LNPCPs, the most common benign etiologies include prior forceps biopsies, endoscopic tattooing in proximity to the lesion site, and adenoma recurrence following p-EMR, all of which trigger the development of submucosal fibrosis [70,71].

Effective management relies on a structured algorithm centered on optical diagnosis. Endoscopists must avoid relying solely on tactile feedback and instead prioritize advanced imaging classifications using VCE. Lesions exhibiting amorphous surface patterns and loss of vessel structure (JNET Type 3) confirm deep SMI and necessitate surgical referral. Conversely, an NLS retaining a regular vascular pattern (JNET Type 2A) or mild irregularity (JNET Type 2B, Kudo Vi) likely represents benign fibrosis and remains amenable to advanced endoscopic treatment [28].

Once the diagnosis of a benign non-lifting lesion is established, the therapeutic goal is curative resection. ESD is the gold standard for non-lifting LNPCPs, allowing for direct visualization and sharp dissection of the fibrotic plane and enabling precise separation of the polyp from the muscularis propria [41]. For benign non-lifting lesions that are so densely adherent that standard dissection is unfeasible, removing the entire bowel wall becomes the only endoscopic option. EFTR offers a definitive solution by removing the full thickness of the bowel wall using a dedicated clip-and-cut device, demonstrating high technical success rates and R0 resection [43]. However, the cap restricts the lesion size to approximately 25–30 mm, limiting its application for larger lesions. To overcome this limitation, combined approaches such as the hybrid EFTR techniques (ESD-EFTR or EMR-EFTR) are emerging. Although current evidence in the literature remains limited, preliminary results appear promising. Notably, Meier et al. highlighted the potential of this strategy for managing extensive lesions characterized by focal fibrosis; this technique involves “debulking” the peripheral lifting portions via EMR, thereby reducing the target lesion to a size that is compatible with the device for the subsequent definitive full-thickness resection of the central fibrotic anchor [72].

### 3.14. What Is the Recommended Post-Resection Follow-Up for Large Non-Pedunculated Colorectal Polyps?

Defining an appropriate surveillance strategy following the removal of LNPCPs is as critical as the resection itself. According to the ESGE guidelines, the surveillance protocol is dictated by the resection technique (piecemeal vs. *en bloc*) and histological risk stratification [24]. For LNPCPs (>20 mm) that are removed via p-EMR, the risk of local recurrence is substantial. A pivotal meta-analysis of 33 studies reported a recurrent adenoma rate of 20% after p-EMR of non-pedunculated colorectal lesions [73]. While complementary techniques such as MTA significantly reduce this risk, they do not eliminate it entirely, as histological confirmation of margin clearance is not feasible [58,74]. Consequently, ESGE strongly recommends a first surveillance colonoscopy (SC1) at 3–6 months following p-EMR of lesions ≥ 20 mm [24]. This short interval allows for the detection and treatment of recurrent adenomatous tissue while it remains diminutive and easily manageable [56,62].

Conversely, if the lesion is removed *en bloc* and pathology confirms free margins (R0), the risk of local recurrence is minimal (<1%); guidelines therefore suggest that early surveillance is not required for these [58]. ESGE generally recommends that patients with complete removal of adenomas ≥ 10 mm or with high-grade dysplasia follow a 3-year surveillance interval. However, expert consensus often favors a more cautious 12-month surveillance for large lesions, particularly those with high-grade dysplasia or superficial submucosal invasion, to confirm healing and rule out metachronous lesions [25,75]. In this context, risk stratification tools such as the Sydney EMR Recurrence Tool (SERT) can further guide surveillance. The Sydney EMR Recurrence Tool (SERT) identifies key predictors of recurrence: lesion size ≥ 40 mm, intraprocedural bleeding, and the presence of high-grade dysplasia [76]. Lesions with a SERT score of 0 have a significantly lower recurrence risk than those with scores of 1–4, potentially allowing for tailored surveillance intervals.

At the first surveillance visit, accurate identification and assessment of the scar are the primary objectives. Tattoos placed during the index procedure (distal to the lesion) are crucial for relocalization. High-definition white light (HD-WL) combined with VCE (NBI, BLI) and near-focus (NF) represents the gold standard for assessment, and routine biopsy of a macroscopically normal scar is not necessary if high-confidence optical diagnosis confirms the absence of neoplasia. Biopsy should be reserved for cases where the optical diagnosis is low-confidence or if the endoscopist lacks experience in advanced imaging characterization [77,78]. If SC1 is negative for recurrence, a second surveillance colonoscopy (SC2) is recommended 12 months later (i.e., 18 months post-resection) to detect late recurrences. If SC2 is negative, the patient can return to standard surveillance intervals (e.g., 3–5 years). Conversely, if recurrence is detected and treated at SC1, an early follow-up (e.g., 6–12 months) is required until clearance is confirmed [25].

## 4. Discussion

LNPCPs pose a significant challenge in daily endoscopic practice, combining a distinct malignant potential with a non-negligible risk of technical failure and adverse events if managed outside a structured pathway. Over the past decade, the therapeutic paradigm has progressively shifted from a surgical-first approach to organ-preserving endoscopic resection, predicated on accurate lesion assessment, operator expertise, and appropriate settings. In this context, the major determinant of optimal outcomes is not the availability of a single “best” technique, but the ability to integrate high-quality detection, reliable optical diagnosis, precise prediction of SMI, and a tailored resection strategy.

The present question-based synthesis underscores that diagnostic quality is a prerequisite for therapeutic success. High mucosal visibility, strict adherence to quality metrics, and systematic “second-look” maneuvers are paramount to reducing miss rates for flat and serrated lesions, particularly in the right colon. Beyond detection, standardized lesion description—size, morphology, and location—must be improved to mitigate common pitfalls such as size misestimation and inconsistent application of the Paris classification. Practical solutions include the use of objective reference tools and structured reporting, which ultimately enhance communication and ensure appropriate referral.

Accurate risk stratification for deep SMI is the cornerstone of the therapeutic algorithm. Morphological “red flags” (e.g., depression, ulceration, fold convergence) and advanced imaging classifications (JNET and pit pattern analysis) must guide the endoscopist toward the correct decision-making process: *en bloc* resection for suspected superficial SMI versus piecemeal resection for lesions lacking invasive features. Crucially, routine biopsy of LNPCPs is strongly discouraged; it is frequently misleading and may compromise subsequent resection by inducing fibrosis and the NLS. Tissue sampling should be reserved exclusively for lesions with endoscopic features that are strongly suggestive of deep SMI when histological confirmation is required before surgery.

Regarding technique selection, EMR remains the standard of care for the vast majority of LNPCPs with a low risk of SMI, offering an excellent balance between efficacy, safety, and resource utilization. Conversely, ESD should be selectively employed for lesions in which accurate histopathological staging and curative intent require an *en bloc* R0 specimen, particularly when superficial invasive cancer is suspected or when the morphology indicates a heightened invasion risk (e.g., LST-NG pseudo-depressed or lesions with a depressed component). Hybrid ESD and alternative strategies can complement the therapeutic armamentarium: underwater EMR may improve outcomes in selected size ranges, while traction-assisted and pocket-creation approaches can increase ESD feasibility and safety and potentially flatten the learning curve. However, these techniques must be applied within an evidence-based strategy rather than used as interchangeable shortcuts, especially when oncologic assessment is the priority.

A recurring theme throughout this review is that clinical outcomes are heavily dependent on procedural planning and expertise. The “see-and-treat” approach during standard diagnostic slots is often inappropriate for complex LNPCPs; dedicated therapeutic sessions, advanced equipment, and a team trained in adverse event management are essential to maximize single-session success and minimize recurrence. Objective tools such as the SMSA score support rational triage by differentiating lesions that are suitable for local management from those requiring referral to expert centers or multidisciplinary discussion. Parallel to this, competence must be defined not only by training thresholds but also by measurable KPIs, including complete resection rates, recurrence rates, and complication benchmarks.

**Figure 2:**
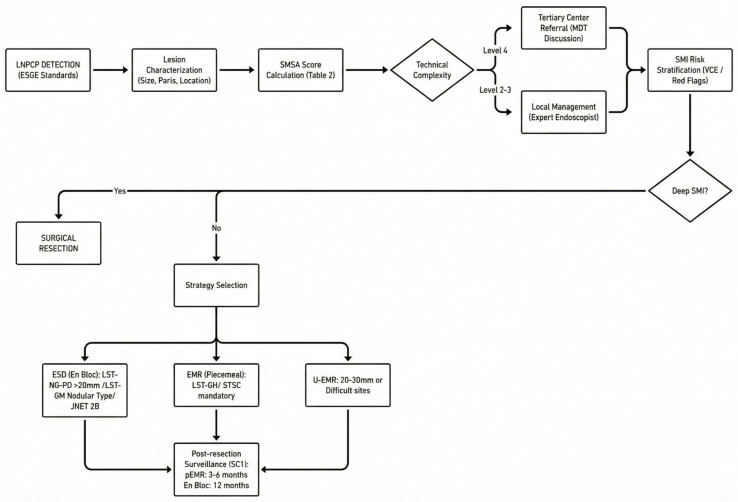
Proposed diagnostic and therapeutic algorithm for LNPCP management. The flowchart integrates detection standards, complexity assessment (SMSA score), risk stratification for submucosal invasion, technique selection (EMR vs. ESD vs. surgery), and post-resection surveillance strategies. LNPC, large non-pedunculated colorectal polyp; SMSA score, size morphology site access score; MDT, multidisciplinary team; SMI, submucosal invasion; VCE, virtual chromoendoscopy; EMR, endoscopic mucosal resection; ESD, endoscopic submucosal dissection; U-EMR, underwater endoscopic mucosal resection; LST-GH, laterally spreading tumor—granular homogeneous, LST-NG, laterally spreading tumor—non-granular; LST-NG-PD, laterally spreading tumor—non-granular pseudo-depressed; LST-GM, laterally spreading tumor—granular mixed; STSC, snare tip soft coagulation; SC1, first surveillance colonoscopy.

Finally, post-resection surveillance is not ancillary but integral to effective LNPCP management. Surveillance intervals must be tailored to the resection technique and histologic risk profile, with early site checks following piecemeal resection to detect and treat recurrence while it remains diminutive and manageable. High-definition endoscopy combined with virtual chromoendoscopy enables accurate scar assessment, reducing the need for routine biopsies when optical confidence is high, while structured follow-up pathways help prevent both under- and over-surveillance. To synthesize the key decision points discussed throughout this review—from initial detection to post-resection surveillance—we propose a comprehensive management algorithm, illustrated in Figure 2.

Our review has some limitations that should be acknowledged. First, as a narrative review, it provides a qualitative synthesis of the evidence rather than a quantitative meta-analysis; consequently, specific pooled estimates for outcomes such as recurrence rates or adverse events were not generated. Second, although a systematic search strategy involving three independent authors was employed, the selection of studies and the formulation of clinical questions inherently carry a degree of subjectivity compared to a systematic review strictly following the PRISMA guidelines. Finally, the included literature on LNPCPs is characterized by significant heterogeneity in study design, definitions of “expert” centers, and differences between Western and Eastern practice, which may influence the generalizability of certain recommendations, particularly regarding advanced techniques like ESD.

In conclusion, the optimal management of LNPCPs requires a structured, evidence-based, and pragmatically organized approach that bridges the gap between guidelines and real-world practice. By translating the current literature into practice-oriented questions and actionable answers, this review provides a framework to standardize lesion assessment, guide appropriate technique selection, support referral decisions, and refine surveillance strategies. Wider adoption of such a pathway has the potential to improve clinical outcomes, reduce recurrence and procedure-related adverse events, and maximize organ preservation by avoiding unnecessary surgery, while ensuring that patients with invasive disease are promptly directed to the most appropriate curative treatment.

## Figures and Tables

**Table 1:** Management of large non-pedunculated polyps of the colon: practice-oriented answers to clinical questions.

Clinical Question	Key Recommendation
** How should LNPCPs be detected? **	Adhere to ESGE quality standards (cecal intubation rate > 90% and withdrawal time > 6 min). Use dynamic maneuvers (retroflexion, second-look) and consider AI (CADe) to minimize miss rates.
** What are common mistakes in LNPCPs assessment? **	Avoid rounding the lesion size to the nearest 10 or 5 mm (“terminal digit bias”); when available, use objective tools (VSE, AI) for lesions > 20 mm to avoid underestimation. Accurately describe location and morphology in the endoscopic report.
** When to suspect deep SMI in LNPCPs? **	**Red flags:** surface depression (Paris 0-IIc), ulceration, fold convergence. **Optical diagnosis:** JNET Type 3, NICE Type 3, Kudo Vn. **Signs:** the NLS has low sensitivity and variable specificity (malignant infiltration vs. benign fibrosis).
** Should large LNPCPs be biopsied? **	Routine biopsy is contraindicated. It causes fibrosis and hinders subsequent endoscopic resection. Exception: perform targeted biopsy only if there is a strong suspicion of cancer (e.g., JNET 3) to confirm diagnosis prior to surgery.
** When to schedule LNPCPs in a dedicated session? **	Avoid “see-and-treat” approaches during standard diagnostic slots (e.g., 45 min). If complexity exceeds the available time, reschedule for a dedicated slot to avoid incomplete resection.
** How to predict technical difficulty in the resection of LNPCPs? **	Calculate the SMSA score to grade complexity. SMSA Level 3 (Score 10–11): requires a dedicated resection session. SMSA Level 4 (Score > 12): consider referral to an expert center or MDT discussion.
** What expertise is required for LNPCP resection? **	Volume: >70 large resections/year. Performance targets: technical success > 91%, recurrence < 16%, bleeding < 19%.
** When is EMR the preferred approach for LNPCPs? **	First line for LST-G and LST-NG < 30 mm without depression. LST-GM: EMR only if dominant nodule can be removed *en bloc*.
** When is ESD the most appropriate approach for LNPCPs? **	First line when suspected superficial SMI (T1a cancer), i.e., LST-GM, LST-NG-PD.
** Is there a role for hybrid/simplified techniques for LNPCPs? **	Consider *en bloc* Underwater EMR for 20–30 mm lesions. Traction/Pocket ESD can be useful to reduce the learning curve and procedure time. Hybrid ESD is oncologically inferior to standard ESD; use mainly as a rescue technique.
** How does LNPCPs’ location influence endoscopic resection? **	Rectum: ESD preferred (lower perforation risk, higher impact of surgery/stoma). Right colon: EMR preferred (thinner wall, higher perforation risk with ESD). Difficult sites (e.g., diverticulum, appendix): consider U-EMR or cap-assisted EMR.
**What are frequent technical errors in LNPCP endoscopic resection?**	Avoid static injection; use dynamic injection with viscous fluid (e.g., glycerol, starch) to maintain the cushion. Avoid blind coagulation of bleeding points (high risk of thermal injury and delayed perforation). Omission of STSC on margins is a critical error and increases recurrence.
** How to manage non-lifting LNPCPs? **	**Differential diagnosis:** crucial to distinguish benign fibrosis (e.g., previous biopsy/tattoo) from malignant infiltration (check for JNET 3). **Treatment:** if benign fibrosis is confirmed, treat with ESD or EFTR.
** What is the recommended follow-up after LNPCP resection? **	**Piecemeal EMR:** SC1 at 3–6 months. ***En Bloc* R0 resection:** SC1 at 12 months. **Method:** inspect scar using HD-WL and VCE. Perform biopsy only if recurrence is suspected/doubtful.

LNPCPs, large non-pedunculated colorectal polyps; AI, artificial intelligence; CADe, computer-aided detection; VSE, virtual scale endoscope; NLS, non-lifting sign; JNET, Japan NBI expert team; SMSA score, size morphology site access score; MDT, multidisciplinary team; LST-G, laterally spreading tumor—granular, LST-GM, laterally spreading tumor–granular mixed; LST-NG, laterally spreading tumor—non-granular; LST-NG-PD, laterally spreading tumor—non-granular pseudo-depressed; EMR, endoscopic mucosal resection; ESD, endoscopic submucosal dissection; STSC, snare tip soft coagulation; SC1, first surveillance colonoscopy; HD-WL, high-definition white light; EFTR, endoscopic full-thickness resection; VCE, virtual chromoendoscopy.

**Table 2:** Recommended management of LNPCPs according to predicted complexity of endoscopic resection based on the SMSA (Size, Morphology, Site, Access) scoring system.

Level	Score Range	Complexity Description	Recommended Setting
Level 1	4–5 points	**Low complexity** Standard polypectomy competencies required	Standard list
Level 2	6–9 points	**Moderate complexity** Requires competency in EMR; generally manageable in routine practice	Standard list or dedicated session based on endoscopist experience
Level 3	10–12 points	**High Complexity** Significant technical challenges; increased risk of incomplete resection	Dedicated therapeutic session is strongly recommended
Level 4	>12 points	**Extreme Complexity** Highest risk of technical failure, adverse events, and recurrence.	Mandatory dedicated session or referral to a tertiary center

EMR, endoscopic mucosal resection.

## Data Availability

No new data were created or analyzed in this study.

## Abbreviations

The following abbreviations are used in this manuscript:

ADR	adenoma detection rate
AI	artificial intelligence
AO	appendiceal orifice
CADe	computer-aided detection
C-EMR	conventional endoscopic mucosal resection
C-ESD	conventional endoscopic submucosal dissection
CRC	colorectal cancer
EFTR	endoscopic full-thickness resection
EMR	endoscopic mucosal resection
ESD	endoscopic submucosal dissection
H-ESD	hybrid endoscopic submucosal dissection
IRR	incomplete resection rate
IV	ileocecal valve
KPIs	key performance indicators
LNPCP	large non-pedunculated colorectal polyp
LST	laterally spreading tumor
LST-G	laterally spreading tumor—granular
LST-GH	laterally spreading tumor—granular homogeneous
LST-GM	laterally spreading tumor—granular mixed
LST-NG	laterally spreading tumor—non-granular
LST-NG-PD	laterally spreading tumor—non-granular pseudo-depressed
MTA	margin thermal ablation
NLS	non-lifting sign
PC-ESD	pocket-creation endoscopic submucosal dissection
p-EMR	piecemeal endoscopic mucosal resection
SMI	submucosal invasion
STSC	snare tip soft coagulation
T-ESD	traction-assisted endoscopic submucosal dissection
U-EMR	underwater endoscopic mucosal resection
VCE	virtual chromoendoscopy
VSE	virtual scale endoscope
